# Validating Electronic Medical Record Documentation of Rejection of Care: The EMRoC Protocol

**DOI:** 10.1093/geroni/igaf122.1339

**Published:** 2025-12-31

**Authors:** Clarissa Shaw, Gregory Issac, Lisa Weimar, Caitlin Ward

**Affiliations:** University of Iowa College of Nursing, Iowa City, Iowa, United States; University of Minnesota, Minneapolis, Minnesota, United States; University of Iowa, Iowa City, Iowa, United States; University of Minnesota School of Public Health, Minneapolis, Minnesota, United States

## Abstract

Rejection of care (RoC) in people living with dementia can lead to harmful practices including physical and chemical restraints. While the Resistiveness to Care scale (RTC-DAT) is the gold standard for measuring RoC through trained observation of 13 behaviors during care encounters, a more pragmatic measurement approach is needed. This study aimed to validate electronic medical record (EMR) nurse charting data against RTC-DAT scores to develop the EMRoC protocol. Eighty-eight care encounters from 16 hospitalized patient with dementia were observed and scored using the RTC-DAT. EMR flowsheet data was extracted for the corresponding observation days, yielding 35 unique shifts in which 24 (68.6%) included RoC. Analysis identified 16 EMR variables that demonstrated complete concordance with the RTC-DAT, successfully classifying 12 of 35 shifts. The EMRoC decision tree was developed to categorize the remaining 23 shifts. The EMRoC was then applied to a large dataset from one hospital spanning 4-years of nurse flowsheet data, encompassing 1,503 patients across 2,141 admissions and 31,598 shifts. Multiple EMRoC configurations were evaluated using area under the curve (AUC) analysis and assessed against data completeness to determine optimal performance. The final EMRoC protocol included 20 EMR variables (16-variables meeting complete concordance and a 4-variable decision tree) and achieved an in-sample accuracy of 91.4% identifying RoC in 57.9% (N = 18,282) of shifts. The validation process revealed which specific nurse-charted EMR variables indicated RoC presence or absence. This study provides a methodological framework for validating nurse flowsheet data against gold-standard tools and offers a practical approach to measuring RoC.

